# Artificial Intelligence Algorithm-Based Economic Denial of Sustainability Attack Detection Systems: Cloud Computing Environments

**DOI:** 10.3390/s22134685

**Published:** 2022-06-21

**Authors:** Theyazn H. H. Aldhyani, Hasan Alkahtani

**Affiliations:** 1 Applied College in Abqaiq, King Faisal University, P.O. Box 400, Al-Ahsa 31982, Saudi Arabia; 2 College of Computer Science and Information Technology, King Faisal University, P.O. Box 400, Al-Ahsa 31982, Saudi Arabia; hsalkahtani@kfu.edu.sa

**Keywords:** machine learning approaches, deep learning approaches, economic denial of sustainability attack, cloud computing, intrusion detection system

## Abstract

Cloud computing is currently the most cost-effective means of providing commercial and consumer IT services online. However, it is prone to new flaws. An economic denial of sustainability attack (EDoS) specifically leverages the pay-per-use paradigm in building up resource demands over time, culminating in unanticipated usage charges to the cloud customer. We present an effective approach to mitigating EDoS attacks in cloud computing. To mitigate such distributed attacks, methods for detecting them on different cloud computing smart grids have been suggested. These include hard-threshold, machine, and deep learning, support vector machine (SVM), K-nearest neighbors (KNN), random forest (RF) tree algorithms, namely convolutional neural network (CNN), and long short-term memory (LSTM). These algorithms have greater accuracies and lower false alarm rates and are essential for improving the cloud computing service provider security system. The dataset of nine injection attacks for testing machine and deep learning algorithms was obtained from the Cyber Range Lab at the University of New South Wales (UNSW), Canberra. The experiments were conducted in two categories: binary classification, which included normal and attack datasets, and multi-classification, which included nine classes of attack data. The results of the proposed algorithms showed that the RF approach achieved accuracy of 98% with binary classification, whereas the SVM model achieved accuracy of 97.54% with multi-classification. Moreover, statistical analyses, such as mean square error (MSE), Pearson correlation coefficient (R), and the root mean square error (RMSE), were applied in evaluating the prediction errors between the input data and the prediction values from different machine and deep learning algorithms. The RF tree algorithm achieved a very low prediction level (MSE = 0.01465) and a correlation R^2^ (R squared) level of 92.02% with the binary classification dataset, whereas the algorithm attained an R^2^ level of 89.35% with a multi-classification dataset. The findings of the proposed system were compared with different existing EDoS attack detection systems. The proposed attack mitigation algorithms, which were developed based on artificial intelligence, outperformed the few existing systems. The goal of this research is to enable the detection and effective mitigation of EDoS attacks.

## 1. Introduction

Cloud computing includes paying only for the services used, renting hardware and software, and more. It refers to the delivery of computer hardware and software, and other information technology services, to a customer or client across a network, depending on their individual requirements [1]. Third-party cloud computing service providers often provide such services by investing in or owning their own data center or network infrastructure. Cloud computing has also emerged as a rapidly expanding division of the information technology industry [2]. However, security remains a significant source of concern regarding this growing technology. Economic denial of service (EDoS) attacks on cloud infrastructure are rapidly becoming difficult security concerns [3,4]. The term economic denial of sustainability was coined by Hoff and Cohen in 2008 [5,6]. Cohen further defined it in 2011 [7], which is now widely accepted by the scientific community. EDoS attacks are typically targeted at cloud computing infrastructures, which are becoming increasingly important in emerging communication technologies. As a result, Singh et al. [8] explicitly defined DDoS attacks as “threats that try to render the pricing model unsustainable and, as a consequence, make it impossible for a firm to financially use or pay for its cloud-based infrastructure” [9,10]. According to research, EDoS threats are also referred to as reduction of quality (RoQ) threats and fraudulent resource consumption (FRC) attacks. Attackers use computational intelligence methods to take advantage of the “pay-as-you-go” accounting model offered by most cloud computing providers, and their auto-scaling capabilities, to commit these breaches.

**Figure 1:**
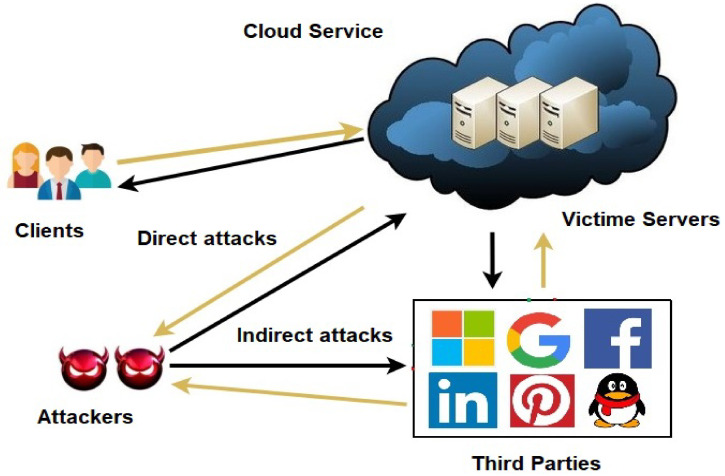
Economic denial of sustainability threat to cloud computing service providers.

The EDoS attack is a new type of distributed denial of service (DDoS) attack. However, unlike a DDoS attack, which might block legitimate customers from accessing a service for a specified period of time, an EDoS attack potentially prevents a cloud adopter from offering services indefinitely, resulting in insolvency [11]. They are more difficult to detect than DDoS attacks because they are relatively new and complicated. Figure 1 illustrates the EDoS threat to cloud computing in both direct and indirect ways. The auto-scaling function of cloud computing is exploited by EDoS attacks, resulting in the creation of new virtual machines that are not needed. The cloud service provider is saddled with the expenses of this unauthorized malevolent use. A progressive increase in illegal traffic is achieved by EDoS attackers. Detecting EDoS attack traffic is difficult since it resembles normal traffic.

Various information security organizations have issued warnings regarding this problem [12,13,14]. For example, DDoS threads have increased by 30% in the last year, according to the European Union Agency for Network and Information Security (ENISA) [15,16]. Several countries, including Europe and the United States [17], and the European Commission (EC), have announced considerable increases in their efforts to resist these assaults because of the severity of threads discovered in Autumn 2016 [18,19]. There are several factors that contribute to their damaging power, according to the European Police Organization (Europol). Some of these include the rapid proliferation of botnets, the emergence of novel vulnerabilities and amplifying elements, the greater availability of malicious products such as Crimeware-as-a-Service on the black market, the widespread use of certain technologies (e.g., mobile devices and the Internet of Things (IoT), and a general lack of awareness among users about cybersecurity best practices.

Different strategies have been proposed to deal with the challenge of EDoS detection. Two machine learning techniques, SVM and self-organizing map (SOM), have successfully identified DDoS attacks [20,21]. However, standard machine learning algorithms have difficulty digesting large EDoS data in network applications since they rely heavily on feature engineering and selection. It is possible to use deep learning (DL), a type of machine learning technology that uses neural layers, to better extract information, increase detection accuracy and robustness, and overcome the limitations of machine learning (ML). DL algorithms make use of a range of neural network models designed to mimic the human brain, and many nonlinear processing units, to cope with challenging problems. For many models and processes that use flow-based detection techniques, it is necessary to have a time series model that can recall the most recent input and predict the output of the sequence data effectively. The recurrent neural network (RNN) can be used to detect DDoS attacks. A memory gate is an inherent mechanism in the long short-term memory (LSTM) RNN model that may be used to address the vanishing gradient problem in RNNs by controlling the flow of input sequences [22].

In comparison to network systems, cloud computing offers certain unique characteristics, including dynamic resource assignment and usage-based billing. Auto-scaling features of cloud services are used by EDoS attacks to inflate the bills of a cloud user to the point where the account is insolvent or large-scale service withdrawal occurs. An EDoS attack uses the economies of scale afforded by the cloud to disrupt or interrupt cloud services and infrastructure, which in turn disrupts or interrupts a company’s applications, systems, and network. Therefore, it is necessary to develop a system based on machine learning and deep learning algorithms to mitigate and detect the EDoS attack. We tested a number of machine learning and deep learning methods, including support vector machines (SVM), K-nearest neighbors (KNN), random forest (RF) tree techniques, and long short-term memory (LSTM). Next, we used the correlation coefficient approach to examine the features of an EDoS attack. In our investigation, we found that the SVM and RF algorithms achieved superior accuracy, and the obtained results ware compared with different existing systems.

## 2. Contribution

EDoS is a type of DoS attack that focuses on the financial aspect of the targeted service. In cloud computing, the EDoS attack forces an unduly large scale-up of the service, resulting in a waste of the cloud resources and money. These attacks have many features of classic DoS attacks, particularly those focused on flooding; however, they typically generate a smaller volume of traffic than traditional DoS attacks. DDoS is a low-frequency DoS attack that is difficult to detect using methods that analyze traffic volume. Therefore, developing a smart system based on ML and DL that can help to distinguish between normal and abnormal network packet dynamics is the main contribution of the proposed study. We investigated the proposed system using real network traffic containing EDoS attacks. The proposed study carried out genuine detection of these attacks in cloud computing environments. The results of this research were compared with different existing systems to prove this system’s robustness and effectiveness.

## 3. Study Background

Most current research on DDoS prevention focuses on approaches for stopping malicious traffic at the network level or application layer. Most cloud computing (CC) and network experts believe that the analysis of network traffic depends on the chosen threshold and entropy [23,24,25,26]. Known EDoS defense strategies include the following.

EDoS attacks may be detected using statistical approaches, such as entropy and fuzzy methods [27]. The detection accuracy is excellent [28]. Even though, they have been tested on a very modest testbed, there are reasons to mistrust the method’s performance in real-world situations. Because of the specified criteria, fuzzy entropy-based EDoS mitigation has significant inaccuracies.

Masood et al. [29] developed EDoS Armor, a cost-effective EDoS attack mitigation framework for cloud-based e-commerce apps. Their study outlined a multitiered defensive strategy to combat an attack. An initial restriction on connections was put in place so that the attack did not become too overwhelming. Then, a technique based on browsing behavior was used to determine user priority. The higher the priority, the more resources were allotted, whereas the lower the priority, the less resources were allotted. This priority value was used to distribute resources across users.

Baig et al. [30] provided a method for restricting access to virtual resources to protect cloud infrastructures from DoS attacks. Their article offered a way to regulate customers’ service demands. This method divided incoming user requests into two categories: regular and suspicious. To ensure that only legitimate and normal users had access to cloud services, additional analysis was carried out. This ensured that those in the suspicious category had lower priority for service access until they were removed from the suspicious list.

Koduru et al. [31] also suggested using detection methods for EDoS. To identify HTTP EDoS attacks, they employed the Time Spent on a Web Page (TSP). When an assault occurs, the TSP is significantly different from the average TSP of a web page under normal circumstances.

Spoofed IP EDoS attacks may be detected and mitigated using a method developed by Al-Haidari et al. [31], which makes use of the IP header’s Time to Live (TTL) field. The researchers used the white/blacklist technique. They employed a verifier and a threshold system to determine whether incoming packets were legitimate or suspicious and then collected source IP addresses and TTL values to create a whitelist and a blacklist for the two types of packets.

Kumar et al. [32] suggested using an architecture to counteract EDoS assaults on online services. To identify a valid user, a crypto puzzle (client puzzle) was created. Customers must answer this conundrum to be granted access to the cloud. Their work was based on the provider evaluation of the system status, which might be normal or suspect, depending on the server and bandwidth demand. The architecture makes a judgment about the difficulty of the challenge based on these data.

To protect cloud computing systems from DDoS assaults, Alosaimi et al. [33] proposed an enhanced DDoS mitigation system (Enhanced DDoS-MS). The first packet received from a user was tested using a Graphical Turing Test (GTT) to differentiate authentic from fraudulent users. Malware may potentially be detected in packets using an intrusion prevention system (IPS). EDoS attacks were mitigated using crypto puzzles and white/blacklists, as in earlier studies [34].

Some researchers have used simple machine learning (ML) approaches, such as selecting an algorithm and training it using a whole dataset. For example, in [35], a multilayer perceptron (MLP) network was used to identify and anticipate harmful assaults on large datasets. Covariance, standard deviation, and correlation were used to identify the characteristics in this scenario. With precision of 0.9935, the authors were able to identify traits that had an almost perfect association.

In the work of Larriva-Novo et al. [36], a cybersecurity dataset was categorized using a static method based on the MLP. This study was conducted to find the optimal hyperparameters for accuracy. Additional criteria used in the selection process included the type of connection, type of content, traffic statistics, and the direction of traffic. The model had anomaly detection accuracy of approximately 99%.

In the design of an intrusion detection system (IDS) based on anomalies, the SVM method is often used to forecast whether or not the incoming data are anomalous. The authors of [37] used a nonlinear scaling strategy for data preparation to enhance the outcomes of their system. Binary and multi-class classifications were performed together with accuracy, detection rate, and false positive rate (FPR) assessments. For binary classification, the accuracy was 85.99%, while, for multi-class classification, the accuracy was 75.77%. In [38], the researchers attempted to go further into the classification process using SVMs. The binary gravitational search enhanced the accuracy of the IDS produced by the researchers. Accuracy of 86.62% was achieved without the use of feature selection.

Shaaban et al. [39] used a CNN similar to the human brain. Unlike handmade features, a CNN learns directly from image-like input samples. In addition to the packet-based technique, the flow-based method may identify DDoS and EDoS assaults.

Yin et al. [40] advocated for the use of RNNs for intrusion detection. The RNN was used to classify sequential input in continuous traffic flow. The recurrent model can anticipate the following character input by sequentially calculating the input. The RNN model is memory-constrained, since the features are layered in memory cells. LSTM may also handle RNN memory issues. In prior research [41,42,43], LSTM outperformed RNNs. Using a sequence flow-based technique, LSTM can detect EDoS with high accuracy and minimize extended dependence issues. Activation functions in recurrent gates slow down the scheme’s training and prediction time. These functions also influence multivariate real-time forecasting with lengthy input sequences. According to another publication [44], BiLSTM is a bidirectional RNN that processes sequence inputs in both forward and backward directions using two hidden layers. An output layer is created by combining both concealed levels. BiLSTM runs the inputs in two directions: backward and forward. The LSTM approach, which runs backward, preserves information from the future and employs the two combined hidden states, allowing for the preservation of information from both the past and the future at any one moment. However, since it calculates inputs in two directions, it takes longer to train and forecast than LSTM. A DL-based EDoS detection mechanism was used based on an analysis of the LSTM algorithm and recommendations for defense. Two recently completed in-depth studies on EDoS features were the inspiration for this, since they both used the LSTM and remedied the limitations imposed by the model complexity [45,46].

Machine learning is discussed in a number of review publications [47]. Self-adaptive evolutionary extreme learning is used to identify DDoS attacks in [48]. Automatic identification of neurons in the hidden layer, as well as finding the appropriate crossover operator, are key parts of the strategy. Experiments suggest that the proposed approach improves the accuracy, which is why it was examined. SDN DDoS assaults may be detected with the use of a method described in [49] (SDN). The authors employed DNN to identify DDoS assaults in real time. Results from the experiments reveal that this technology identifies DDoS assaults more accurately and with less resource use in less time. A comparison of machine learning algorithms for DDoS detection was performed by the authors in [50]. DDoS assaults can be more accurately detected by RF, according to the findings of our tests. Using methods including correlation, information gain, and the relief feature selection approach, researchers were able to identify the most useful characteristics for detecting DDoS attacks [51]. In this paper, a comparison of several machine learning approaches is made. An intrusion detection method was developed by Manimurugan et al. [52] to identify irregularities in Internet of Things (IoT) networks. Deep belief networks were employed to identify attacks. The CICIDS 2017 assaults dataset is used in the tests. For typical class classification, the suggested technique has a 99.37 percent success rate and a 96.67 percent success rate for DDoS assault detection. Dehkordi et al. [53] proposed a paradigm for SDN that can identify all DDoS assaults.

DDoS attack detection using an autoencoder and recurrent neural network (RNN) was proposed by Elsayed et al. [54] and achieved an F1 score of 99 percent using binary classification. Using an intrusion dataset, Javaid et al. [55] suggested an autoencoder and softmax regression-based classifier model. To attain accuracy of 88.98 percent, the authors of another paper developed a hybrid technique that used autoencoders and isolation forests [56]. When it comes to CICDDoS2019, the F1 score achieved by Wei and colleagues is 98%. It is common for these studies to focus on a particular dataset in order to evaluate the success of their proposal [57].

To detect general intrusion attacks in the smart energy system, including some aspects of DDoS attacks, Ferrag et al. [58] proposed an RNN-based deep learning model and evaluated their proposal with three different datasets, including the CIC-IDS2017 dataset, a power system dataset, and the Bot-IoT dataset, with accuracy of up to 98 percent. There is an autoencoder model that may be used to guard against DDoS assaults on the smart grid, as proposed by Zhou and colleagues [59]. As opposed to utilizing publicly available datasets, they created their own dataset of 2 million DoS attack records and then evaluated their model, reaching classification accuracy of 96%.

We present an AI-enabled EDoS detection system that can be used in cloud computing environments and can defend against a variety of cybersecurity assaults. The intrusion detection system component of our suggested method provides the security of smart cloud computing against any integrity attacks that seek to change important transport maintenance data. These attacks might come from a variety of sources. The component of our proposed solution that makes use of machine learning and deep learning is able to identify and categorize a wide variety of distributed denial of service attacks, any one of which has the potential to obstruct or halt the transfer of time-sensitive and essential maintenance data across a cloud computing platform.

## 4. Materials and Methods

**Figure 2:**
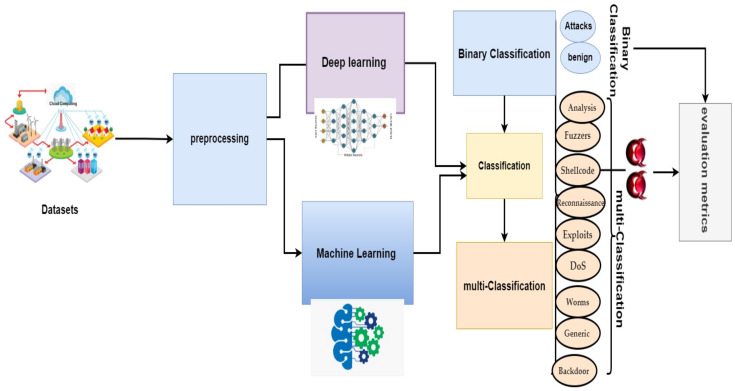
Framework of the proposed system.

The system’s framework for detecting EDoS attacks on cloud computing environments is presented in Figure 2.

### 4.1. Datasets

**Figure 3:**
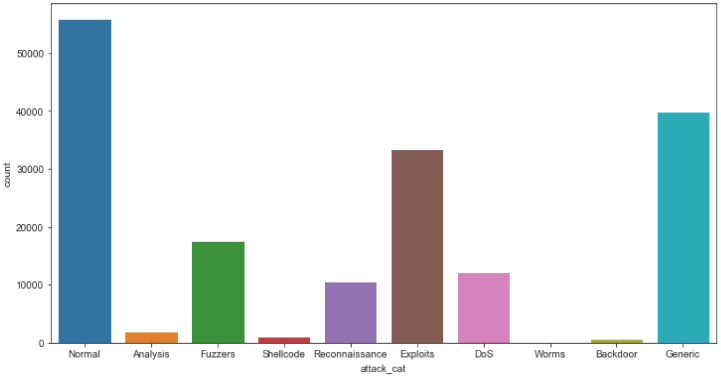
Volume of the dataset for each class.

The IXIA PerfectStorm application was used in the Cyber Range Lab at the University of New South Wales (UNSW), Canberra, to create a blend of true modern routine activities and synthetic contemporary attacks. The PerfectStorm line of load modules offered by Ixia is a scalable solution for testing network security systems for both wired and wireless networks. The tcpdump tool was used to capture 100 GB of raw traffic, which was then analyzed (e.g., Pcap files). This dataset has a number of injection attacks, such as fuzzers, analysis, backdoors, DoS, exploits, generic reconnaissance, shellcode, and worms. The volume of the dataset is presented in Figure 3. This type of malicious software, known as a computer worm, takes advantage of security flaws in a system to steal data and install backdoors that allow others to gain unauthorized access. In addition to taking up a lot of space, worms also take up a lot of bandwidth. A description of the attack dataset is presented in Table 1.

### 4.2. Preprocessing

The dataset had 49 attributes and 175,341 rows; after dropping null values, the dataset had 45 attributes and 80,977 rows. The data type attributes were converted using the data type information from the provided dataset features.

#### 4.2.1. One-Hot Encoding

The one-hot encoding method was used to convert categorical features, namely “protocol”, “service”, “state”, into numerical values, enabling the classifications to detect the attacks.

#### 4.2.2. Min–Max Normalization Method

The normalization process using the min–max method is one of the most used in data normalization. When it comes to each characteristic, the smallest value of the feature is changed to 0, the largest value is transformed into 1, and every other value is transformed into a decimal between 0 and 1 intruders. The min–max normalization method is applied using Equation (1).

(1) $$\overset{´}{Norm}=\frac{norm-y_{min}}{\max\left(B\right)-\;\min\left(B\right)}\;\left(\mathrm{new}\_\max\left(B\right)-\mathrm{new}\_\min(B\right)+\mathrm{new}\_\min(B))$$

where the $\max\;\left(B\right)$ and $\min\;\left(B\right)$ are the minimum and maximum input data that are specified; the new min (B) and new max (B) are the new values of the respective minimum and maximum that were used for the scaling of the data, and Norm is the normalized dataset.

### 4.3. Machine Learning Algorithms

In this section, the theoretical explanations for the ML and DL methods employed in this research are presented. Over the last few years, ML has risen in popularity, and engineers have solved different types of real applications using ML and DL models. SVM, KNN, and RF tree are widely used for detecting intrusions in different network platforms.

#### 4.3.1. Support Vector Machine (SVM)

SVM is one of the most prominent supervised learning algorithms used for classification and regression problems. It is mainly used in ML to solve classification problems. The purpose of using the SVM method is to find the optimal line or decision boundary that can divide n-dimensional space into classes so that fresh data points can easily be classified in the future. The optimal choice boundary is represented by a hyperplane [60]. The method selects the extreme points/vectors that will aid in the creation of the hyperplane in the first place. These extreme points are referred to as support vectors, and the technique for detecting them is referred to as SVM. A nonlinear SVM classifier is used for nonlinearly separated data. This implies that if a dataset cannot be categorized using a straight line, the data are termed nonlinear. In this research, SVM classification with nonlinearity was employed, and the radial basis function (RBF) was used to classify EDoS attacks on cloud computing services.

(2) $$K\left(X,X^{\prime }\right)=exp(-\frac{\|X-X\prime {\|}^{2}}{2\sigma {\;}^{2}\;})$$

where a feature vector used for training an algorithm on a dataset is referred to as X,X″. This feature vector is also used to evaluate the dataset. Moreover, $(X-{X\prime }^{\|2}$) is the squared Euclidean difference between two feature inputs, and it is a variable that can be changed.

#### 4.3.2. K-Nearest Neighbors (KNN)

KNN is one of the simplest and most important classification algorithms in ML. Supervised learning is a widely used technique in pattern recognition, data mining, and intrusion detection. Its lack of underlying assumptions about the distribution of data makes it largely dispensable in real-world circumstances [61,62,63]. The purpose of the KNN algorithm is to assign a class label to a given query point by identifying the nearest neighbors. We observed that a k value of 5 was very appropriate for detecting EDoS attacks.

(3) $$A_{i}=\sqrt{\left(c_{1}-c_{2}\right)+(d_{1}-d_{2}})$$

The k value is used to locate and compute the points on the feature vectors closest to each other. As a result, the value must stand out from the distinctive. Furthermore, $c_{1}-c_{2}$ and $d_{1}-d_{2}$ are feature vectors for finding the closest point.

#### 4.3.3. Random Forest Tree

It is possible to use RF for classification and regression, since it is a supervised learning method. However, it is often used to tackle common classification problems. As is well known, a forest is made up of trees, and more trees equate to a healthier, biodiverse forest. As with the decision tree algorithm, the RF algorithm constructs decision trees from data samples, obtains predictions from each of them, and finally votes to determine which choice is the best. It is an ensemble approach that outperforms a single decision tree because it eliminates overfitting by taking an average of the results. Random refers to the arbitrary selection of input characteristics on each decision tree, using replacement sampling as an input. Repetition of inputs to each decision tree may reduce the algorithm’s accuracy. A large variance problem occurs if a tiny part of the dataset is substituted when predicting results using a decision tree. In RF, a model’s overall prediction may not be affected by various factors from the dataset. RF algorithms outperform decision trees in terms of accuracy [64,65,66,67]. The RF method has been shown to provide greater prediction accuracy because it draws on the outcomes of numerous decision trees to construct forecasts. The most important factor in determining the relevance of features in decision trees and random forests is information gain.

Information gain is a decrease in entropy. The information gain that may be learned about a random variable or signal through the observation of another random variable is referred to as the information gain. The order in which qualities are listed in the nodes of a decision tree may be determined with the use of information gain. The primary node is referred to as the child node, while the subsidiary nodes are referred to as the parent nodes. We are able to evaluate the quality of the splitting of nodes in a decision tree based on the information gain.

### 4.4. Introduction to CNN and LSTM

A CNN is a specific type of multilayer perceptron; however, unlike DL architecture, a basic neural network cannot learn complicated features. Many applications, such as image classification, object identification, and medical image analysis, have demonstrated the superior performance of CNNs [68,69,70,71]. Local features may be obtained from high-layer inputs using CNNs, and these are then transferred to lower layers to be used for more sophisticated features. There are three types of convolutional layers in a CNN: convolution, pooling, and fully connected.

**Figure 4:**
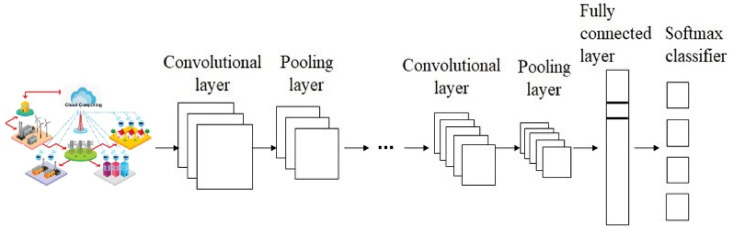
The CNN structure.

The convolutional layer consists of a collection of kernels for calculating the tensors of feature mappings. They convolve a whole input with the help of the “stride(s)” function, such that the dimensions of an output volume are integer numbers [72]. Input volume dimensions shrink when the convolutional layer is used in carrying out striding procedures on the input volume. The zero padding technique is used to pad an input volume with zeros while maintaining the size of an input volume with low-level characteristics, as shown in Figure 4. The CNN parameters are presented in Table 2.

**Figure 5:**
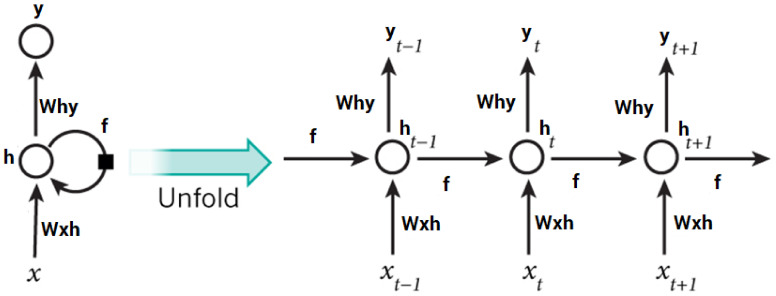
Structure of the LSTM technique.

RNNs are useful for many real-life applications, such as time series forecasting and intrusion detection systems, and are able to find patterns from entire datasets. Artificial neural networks, known as RNNs, were first developed for natural language processing (NLP). RNNs can deal with the difficulties of long-term dependencies in sequential data since they keep the memory of inputs. Consequently, RNNs have a primitive sort of short-term memory and are more effective at detecting short-term patterns in data than conventional feedforward networks. RNNs have improved their ability to store long-term memory [73,74]. When handling the vanishing and exploding gradient issue, LSTM considers using memory blocks instead of standard RNN units. It then adds a cell state to store long-term states, which is its fundamental distinction from RNNs. An LSTM network is able to recall and link data from the past with data from the present. When using LSTM, the input gate is coupled with a “forget” gate, which is used to store the current and previous states of the cell. The output gate is used to store the current state of the cell. Figure 5 depicts the LSTM internal structure.

(4) $$f_{t}=\sigma \left(W_{f\;}.\;X_{t}+W_{f}.\;h_{t-1}+b_{f}\right)$$

(5) $$i_{t}=\sigma \left(W_{i}.\;X_{t}+W_{i\;}.\;h_{t-1}+b_{i}\right)$$

(6) $$\;\;\;S_{t}=tanh\left(W_{c}.\;X_{t}+W_{c\;}.\;h_{t-1}+b_{c}\right)$$

(7) $$C_{t}=(i_{t}*S_{t}+f_{t}*S_{t-1})$$

(8) $$o_{t}=\sigma \left(W_{o}+X_{t}+W_{o}\;.\;h_{t-1}+\;V_{o}\;.C_{t}+b_{o}\right)$$

(9) $$h_{t}=o_{t}+\tan h\left(C_{t}\right)$$

To express the arithmetical notations in the above formulations, the following notations are used:

$X_{t}$ is the vector of the input data that are forwarded to the memory cell at time *t*;
$W_{i}$, $W_{f}$, $W_{c}$, $W_{o}$, and $V_{O}$ refer to the weight matrixes;
$b_{i}$, $b_{f}$, $b_{c}$, and $b_{o}$ are point to bias vectors;
$h_{t}$ indicates the specified value of the memory cell at time *t*;
$S_{t}$ and $C_{t}$ are defined values of the candidate state of the memory cell and the state of the memory cell at time *t*, respectively;
σ and tanh represent the activation functions in the LSTM neural network;
$i_{t}$, $f_{t},$ and $o_{t}$ are the obtained values for the input gate, the forget gate, and the output gate at time *t*, respectively. These gates have values in the range of 0–1 over the nonlinear sigmoid activation function.

### 4.5. Performance Measurements

The evaluation metrics include the computation of the sensitivity, specificity, precision, recall, F1 score, mean square error (MSE), Pearson correlation coefficient (R), and root mean square error (RMSE) to evaluate the effectiveness of the suggested algorithms in identifying EDoS malwares. The following are the equations for the parameters in question:

(10) $$MSE=\frac{1}{n}\;\overset{n}{\underset{i=1}{\sum }}{\left(y_{i,exp}-y_{i,\;pred}\right)}^{2}$$

(11) $$RMSE=\sqrt{\overset{n}{\underset{i=1}{\sum }}\frac{{\left(y_{i,exp}-y_{i,pred}\right)}^{2}}{n}}$$

(12) $$R^{2}\;bn1-\frac{\sum_{i=1}^{n}\;{\left(y_{i,\;exp}-y_{i,\;pred}\right)}^{2}}{\;\sum_{i=1}^{n}\;{\left(y_{i,\;exp}-y_{avg,\;exp}\right)}^{2}\;}$$

#### 4.5.1. Accuracy

The accuracy metric is a helpful assessment metric, but only in situations in which the datasets are consistent and the false positive and false negative values are nearly equivalent to one another. The accuracy of a classifier is measured by how well it is able to predict the data points.

(13) $$\mathrm{Accuracy}=\frac{TP+TN}{TP+FP+FN+TN}\times 100\%$$

#### 4.5.2. Recall

The term “recall” refers to the proportion of correctly predicted positive observations in comparison to the total number of observations made in the actual class. The term “precision” refers to the likelihood that the classifier is making accurate predictions on the real positive class.

(14) $$\mathrm{Sensitivity}=\frac{TP}{TP+FN}\times 100\%$$

#### 4.5.3. Precision

The ratio of the number of correctly predicted positive observations to the total number of expected positive observations is the definition of precision. A low percentage of false positives is typically linked with high levels of accuracy. Precision is a measure of how well the classifier can predict the positive class. It is expressed as a percentage.

(15) $$\mathrm{Precision}=\frac{TP}{TP+FP}\times 100\%$$

#### 4.5.4. F1 Score

The F1 score is calculated by taking the weighted average of the accuracy and recall scores. As a direct consequence of this, this score incorporates both erroneous positive and negative results. Although the F1 score is easier to calculate than accuracy, it is more valuable, especially in cases when there is an uneven distribution of classes. The F1 score is a harmonic mean that combines recall and accuracy.

(16) $$R\%=\frac{n\left(\sum_{i=1}^{n}y_{i,exp}\;\times y_{i,\;pred}\right)-\left(\sum_{i=1}^{n}y_{i,exp}\right)\left(\sum_{i=1}^{n}y_{i,\;pred}\right)}{\sqrt{\left[n{\left(\sum_{i=1}^{n}y_{i,exp}\right)}^{2}-{\left(\sum_{i=1}^{n}y_{i,exp}\right)}^{2}\right]\left[n{\left(\sum_{i=1}^{n}y_{i,pred}\right)}^{2}-{\left(\sum_{i=1}^{n}y_{i,pred}\right)}^{2}\right]}}\times 100$$

When the input data are represented as cloud computing network data (y ($y_{i,exp}$), the experimental value of the data point, i, is represented as y ($y_{i,pred}$), and the predicted value of the data point, i, is represented as y ($y_{avg,exp}$). The average of the experimental values is represented as *y_avg,pred_*, and R, the Pearson correlation coefficient, is represented as y ($y_{i,exp}$); y (*i*, $y_{i,class}$) are the network data classes; and i is the total amount of input data, where in is the total number of input data. TP is the true positive, TN is the true negative, FP is the false positive, and FN is the false negative.

## 5. Experiment

In this section, we present the classification performance of the SVMs, KNN, RF, CNN, and LSTM approaches for evaluation metrics such as accuracy, precision, recall, F1 score, MSE, RMSE, and R^2^. The classification algorithms were analyzed in two scenarios—binary and multi-classification. The binary classification considered a two-class normal or attack dataset. The multi-classification considered a nine-class dataset, namely fuzzers, analysis, backdoors, DoS, exploits, generic, reconnaissance, worms, shellcode, and normal detection, to improve the cloud computing environment against any threat from these attacks.

These two classifications were carried out using a set of 49 features and an ideal set of 45 characteristics. Using correlation algorithms, we were able to identify the ideal collection of 45 features with robustness that correlated with the considered class label. The results of the proposed algorithms were compared with different existing systems. A detailed description of this study is presented in the following subsection.

### 5.1. Experimental Setup

Deep learning approaches such as CNN and LSTM models were implemented using Tensor Flow and Keras, while SVM, KNN, and RF were implemented using Scikit-learn. To analyze the performance, two different categories were used. The software setup was an Intel (R) Core (TM) i7–4770 CPU, 3.20 GHz, 8 GB memory, and running on 64-bit Windows 10. To prevent overfitting in CNN and LSTM, 0.50 dropouts were employed during the training of the model.

### 5.2. Splitting Dataset

The dataset was divided into 70% training and 30% testing. Testing was used to examine the results of the ML and DL approaches. Table 3 shows the sizes of the datasets.

### 5.3. Results of Machine Learning Algorithms

Table 4 shows the performance of the ML algorithms, namely SVMs, KNN, and RF, in detecting EDoS attacks on cloud computing platforms using binary data. The dataset was divided into 70% for training and 30% for testing the model’s ability to detect attacks on the binary datasets. We discovered that the RF tree algorithm achieved the highest testing accuracy of 99% using binary classification. The weighted average of the RF algorithm for detecting EDoS attacks in the testing phase was 99% for each of the measured performance metrics, including precision, recall, F1 score, and accuracy.

**Figure 6:**
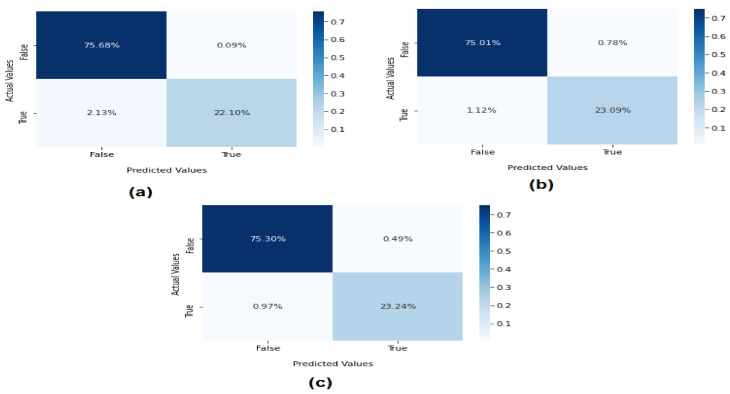
Confusion metrics of machine learning on binary classification: (**a**) SVM, (**b**) KNN, and (**c**) RF.

Figure 6 presents the confusion metrics of ML using binary classification for the detection of EDoS attacks on cloud computing environments. The confusion metrics for evaluating the ML models are reported as true positive (TP), false positive (FP), true negative (TN), and false negative (FN). It was observed that the SVM method scored 75.68% in correctly classifying normal packets. The TP score for correctly classified attacks was 22.10%, while the FP (misclassification) score was 2.13%. The results of the KNN model showed that 75.01% were correctly classified as normal, while the TP score of 23.09% was higher than that of the SVM method. The FP score was very low at 1.12%. Overall, on the binary classification, the RF tree algorithm achieved higher accuracy than SVM and KNN as follows: TN = 75.30% and TP = 23.09%. Meanwhile, the FP was very low at 0.97%. The results show that the RF model can accurately detect and classify EDoS attacks.

Table 5 shows the performance of the SVM algorithm in detecting EDoS attacks on multi-classification datasets. In this experiment, nine-class and normal packets were injected into the datasets to test the proposed ML’s ability to detect malicious attacks. The SVM algorithm achieved high accuracy of 97.56% in the testing phase. 

The results of the KNN algorithm’s ability to detect nine EDoS attacks from the real network dataset are summarized in Table 6. In the evaluation, the testing accuracy of the KNN model was 97.14%. The weighted average for the evaluation metric was 97%.

Table 7 shows the results of the RF tree algorithm for detecting anomalies on service providers’ cloud computing environments. The RF algorithm achieved 97.50% accuracy in detecting nine EDoS attacks under the multi-classification scenario. The performance of the RF algorithm was 0.00 in detecting backdoor attacks. The results for precision, recall, and F1 score in detecting worm attacks were low, at 31%, 11%, and 16%, respectively.

### 5.4. Results of Deep Learning Algorithms

In this section, the results of the DL, CNN, and LSTM models for detecting EDoS attacks in cloud computing environments are presented. Two experiments were conducted on high-performance security systems. The datasets were divided into 70% training and 30% testing.

Table 8 shows the results of the DL models for the binary classification of data, which included two classes of normal or attacks. CNN and LSTM achieved high accuracy metrics of 98.15% and 98.27%, respectively. Overall, both DL models were highly accurate in detecting EDoS attacks.

**Figure 7:**
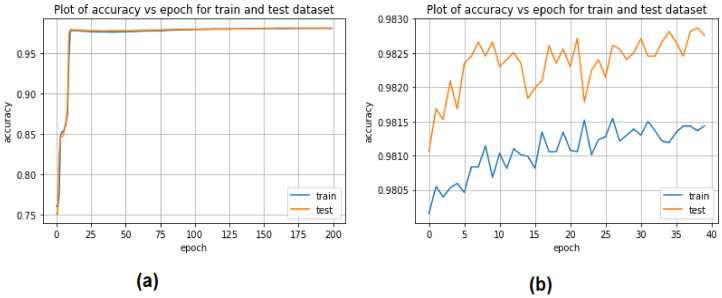
Performance of deep learning models for binary classification: (**a**) CNN, (**b**) LSTM.

Figure 7 depicts the CNN and LSTM strategies for predicting the detection of EDoS attacks on cloud computing environments using a binary classification approach. The accuracy prediction rate of the CNN model started from 75% in the training phase and rose to 98% in the testing phase, while the accuracy of the LSTM model started from 98.10% in the training phase and rose to 98.25% in the testing phase.

**Figure 8:**
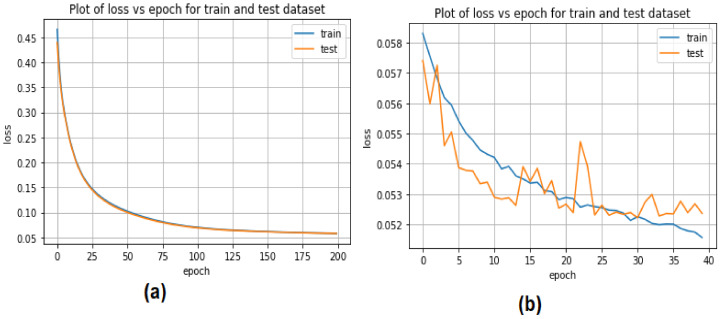
Accuracy loss of deep learning models for binary classification: (**a**) CNN, (**b**) LSTM.

The CNN and LSTM training and testing models for detecting EDoS attacks using binary datasets are presented in Figure 8. The CNN model showed some accuracy in the training and testing phases, decreasing from 0.45 to 0.10 with 20 epochs. The accuracy of the LSTM approach with 20 epochs was low, ranging from 0.057 to 0.053 in the testing stage and from 0.058 to 0.052 in the training phase.

Regarding the second experiment, the results of the DL algorithms on a nine-class dataset are summarized in Table 9. LSTM had a high level of accuracy at 90.35%, while the CNN’s level of accuracy was 84.46%. LSTM’s weighted average in detecting EDoS attacks was 88%, 90%, and 88% for the precision, recall, and F1 score metrics, respectively.

**Figure 9:**
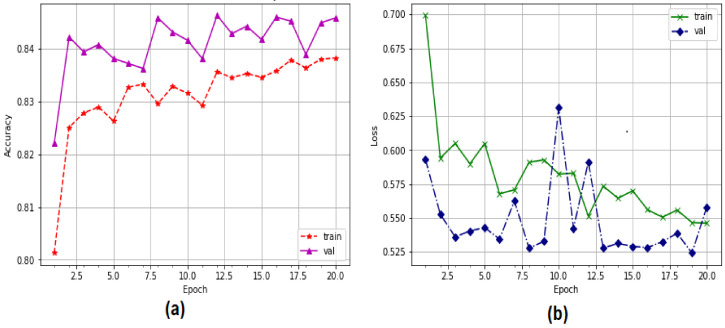
Performance of CNN model using multi-classification: (**a**) accuracy, (**b**) loss.

Figure 9 illustrates the performance validation of the proposed model in recognizing attacks and normal packets during EDoS attack detection tests. The CNN model had validation accuracy of 84.46%, starting from 82% with 20 epochs during an operational period. Using cross-entropy measurements, the validation loss was minimized to as low as 0.77, which is a significant reduction from the original value of 0.54.

**Figure 10:**
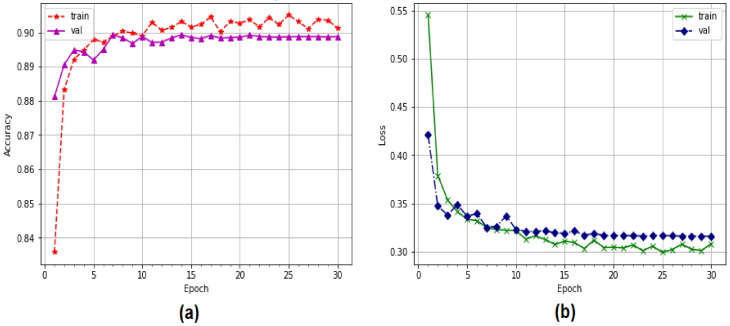
Performance of LSTM model using multi-classification: (**a**) accuracy, (**b**) loss.

Figure 10 depicts the accuracy of the proposed system in terms of precision. The proportion of correctly classified data is shown on the y-axis. The correctness of the training system was determined by the performance of the validation system. We observed an interruption in the system optimization process, enhancing the accuracy to 20 epochs, which is remarkable. The CNN-LSTM model’s performance improved from 88% to 90.35% during the validation process. To calculate the training losses in the proposed system, a categorical cross-entropy function was employed. The LSTM loss is illustrated in Figure 9b. The validation losses dropped from 0.43 to 0.33 with 20 epochs, while the training losses dropped from 0.55 to 0.30 with 20 epochs.

### 5.5. Statistical Analysis

Statistical analysis is the process of gathering and interpreting data to identify patterns and trends. It is part of the data analysis process. Statistical analysis is used in various settings, including data collection, the interpretation of research findings, statistical modeling, and the design of surveys and studies. Moreover, it is beneficial in finding the correlation between the dataset and label features, and finding the errors between the prediction and target values using different measurement metrics. Table 10 shows the statistical analysis of the ML and DL models for the binary dataset. The RF tree algorithm had very low prediction errors compared with the other algorithms according to the Pearson correlation test (R^2^ = 92.02%). RF also had low prediction errors for the MSE and RMSE metrics at 0.0146 and 0.0147, respectively.

Table 11 shows the statistical analysis of ML on a multi-classification dataset. The RF algorithm had very low error levels for MSE and RMSE and a high correlation, with R^2^ = 99%. The RF tree had very low prediction error levels of 0.0576 for MSE and 0.156 for RMSE.

**Figure 11:**
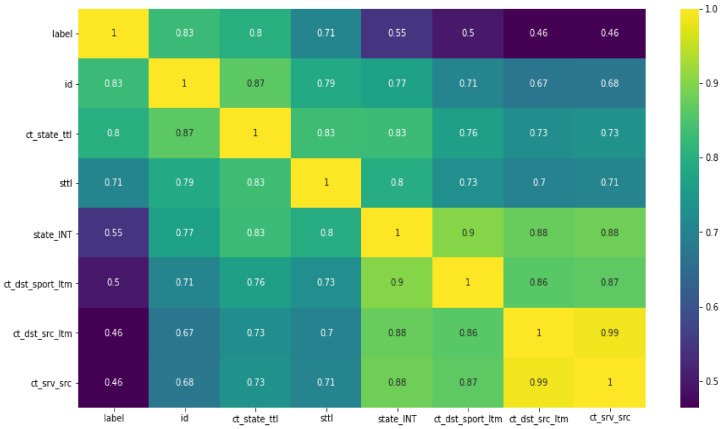
Results of Pearson correlation determining the features with highest correlation labels.

The features that achieved the highest correlation with the nine classes of the dataset are presented in Figure 11. We selected the features that scored greater than 50% in relationships within all nine classes. It was observed that these features were highly correlated with the dataset labels. We considered these features important for detecting EDoS attacks.

## 6. Results and Discussion

The security challenges surrounding cloud computing make it an insecure utility model to use. Using EDoS shielding methods, we attempted to determine whether the source of a request was legitimate or fraudulent. The primary goal of this attempt is to prevent the attacker from depleting the victim’s metered bandwidth, a cloud-specific attack referred to as FRC. To prevent the exploitation of the cloud utility pricing mechanism, intrusions in the form of a DDoS attack are launched. It can be likened to the pricing model employed by utilities, in which customers only pay for the services, such as electricity, that they actually need for daily living. The adversary’s intent is to exploit the victim’s bandwidth for an extended period of time. This bandwidth is provided by the cloud service provider to clients who pay to utilize the service.

Security, on the other hand, is one of the most significant problems preventing the widespread use of cloud computing. These cloud infrastructures continue to be plagued by DDoS attacks, causing widespread devastation. In addition to DDoS attacks, a new type of attack called EDoS has evolved in recent years. When a DDoS attack occurs in a normal computer environment, the service is often disrupted, resulting in financial losses and a negative impact on the client’s reputation. The auto-scalability (elasticity), capabilities, and availability of service level agreements (SLA) in the CC environment make service interruptions very uncommon. Hence, the development of a system that can help to prevent EDoS attacks from threatening the cloud computing environment was the main motivation of this research. The DL, ML, SVM, KNN, and RF tree algorithms, namely CNN and LSTM algorithms, were proposed for classifying and predicting EDoS attacks. The ML and DL algorithms showed good performance levels.

**Figure 12:**
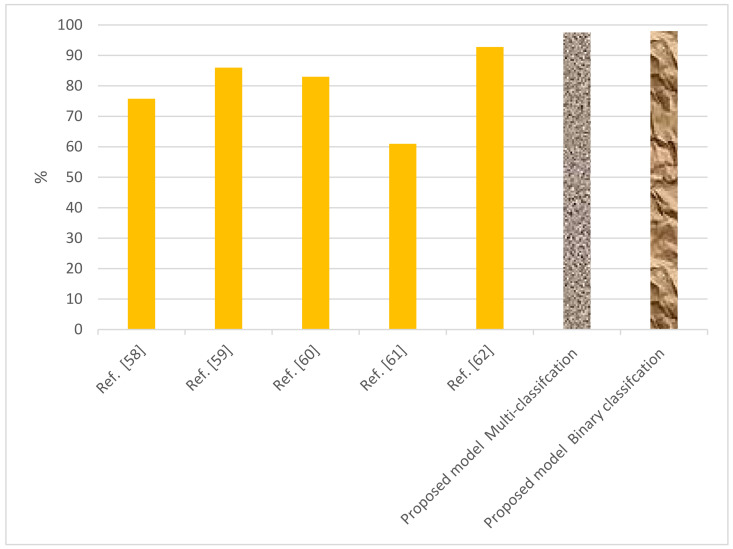
Comparison of performance of the proposed system with different existing systems for detection of EDoS attacks.

We conducted two experiments, namely binary classification and multi-classification, to test these algorithms. The experiments showed that the RF tree had a high score at 99% for binary classification, while the LSTM had a score of 98.27%. In the multi-classification experiments, the SVM model scored 97.56%. A comparative analysis of the proposed ML and DL models is presented in Table 12. It shows the results of the proposed algorithms compared with different existing systems. Therefore, we conclude that our proposed system achieved a high level of accuracy (97.54%) using multi-classification. The RF tree and LSTM also showed high levels of accuracy. Graphical representations of the significant results of the proposed algorithms against different existing algorithms are presented in Figure 12.

## 7. Conclusions

Cloud computing is a breakthrough idea that has revolutionized information and communication technology by offering computational resource services via the Internet, allowing people to use them anywhere in the world. In contrast to traditional computing, cloud computing delivers affordable and scalable on-demand resources for system needs, eliminating the need to purchase and maintain large computer systems. The most significant advantage of cloud computing is the availability of on-demand services at any time. Payment for these services is based on usage charges. As a consequence of this functionality, a new type of DDoS attack, known as EDoS, was created in which the client pays an extra fee to the cloud provider as a result of the attack.

In this study, we evaluated an improved EDoS attack detection and mitigation system based on SVM, KNN, RF tree algorithms, and DL, namely CNN and LSTM. The system design involved real network datasets from the Cyber Range Lab at the University of New South Wales (UNSW), Canberra, which included fuzzers, analysis, backdoors, DoS, exploits, generic, reconnaissance, shellcode, and worms. The results of the present study are promising; consequently, we infer the following:

- The proposed systems are based on ML and DL models for detecting EDoS attacks in cloud computing. The assessment and findings demonstrate that the system is efficient in terms of accuracy.
- We offer two EDoS detection scenarios: binary classification, which contains normal and attack classes only, and multi-classification, which contains nine classes of attacks.
- Statistical analysis was applied to find the percentage of error between the input and prediction values from different ML and DL models.
- Overall, the RF tree demonstrated the best ability to detect EDoS attacks on binary classification, whereas the SVM method had the best ability on multi-classification datasets.
- The experiments revealed that the proposed system produced better results than existing systems.
- It is also noteworthy that the performance of ML models is marginally superior to that of mathematical models. The ML model has precision of 100%, whereas the mathematical model has precision of 99% for binary classification and 97.56% for multi-classification datasets.

## Figures and Tables

**Table 1:** All attack datasets; the dataset has 9 attacks and a normal case.

Attacks	Description
Analysis	A hacker attempts to reach the same network as the user to listen to (and record) network traffic.
Fuzzers	A fuzzing attack is a procedure that is automated and used to identify vulnerabilities in software applications. It involves injecting enormous quantities of random data, also known as fuzz, into a source code and observing the results of the experiment.
Shellcode	Shellcode is a specific type of code that may be remotely inserted and used by hackers to attack a wide range of software vulnerabilities and flaws. It has this name because it usually results in the spawning of a command shell from which attackers may gain control of the vulnerable machine.
Reconnaissance	When an intruder interacts with a targeted system to obtain knowledge on vulnerabilities, it is known as active reconnaissance.
Exploits	The term “exploit” refers to an attack on a computer system, particularly one that takes advantage of a specific weakness that the system makes available to intruders.
DoS	A DoS assault is a type of cyberattack that attempts to bring a computer or network to a halt, rendering it inaccessible to intended users. DoS attacks do this by flooding the target with traffic or by feeding it information that causes the target to crash and shut down.
Worms	One of the fundamental functions of a computer worm is to self-replicate and infect other uninfected computers.
Backdoor	A backdoor is a type of malware that allows users to obtain access to a system by circumventing conventional authentication mechanisms. Therefore, remote access is acquired to resources inside an application, such as databases and file servers, giving offenders the ability to remotely issue system instructions and update malware without the need to physically access the resource.
Generic	It is possible to perform a general attack against a cryptographic primitive without concern for the specifics of how this particular cryptographic primitive was developed.

**Table 2:** Important parameters of the CNN approach.

Parameter Types	Parameter Values
Kernel size value	5
Max pooling size value	4
Dropout layer value	0.50
FC layer	512
Activation function and optimizer operator	ReLU function and Adam
Size of epochs	20
Batch size	50

**Table 3:** Splitting of UNSW datasets.

Variable	Training Size	Testing Size
Dataset	56,683	24,294

**Table 4:** Results of machine learning for binary classification.

Algorithm	Classes	Accuracy (%)	Precision (%)	Recall (%)	F1 Score (%)
SVM	Normal	98	97	100	99
	Attacks		100	91	95
	Weighted Average		98	98	98
KNN	Normal	98	99	99	99
	Attacks		97	95	96
	Weighted Average		98	98	98
RF	Normal	99	99	99	99
	Attacks		98	96	97
	Weighted Average		98	98	98

**Table 5:** Results of SVM on multi-classification.

Attacks	Precision %	Recall %	F1 Score %
Analysis	100	100	100
Backdoor	0.00	0.00	0.00
DoS	100	100	100
Exploits	100	100	100
Fuzzers	47	47	**47**
Generic	99	99	**99**
Normal	100	100	**100**
Reconnaissance	58	65	**61**
Worms	00	0.00	**0.00**
**Accuracy**	97.56%		
**Weighted Average**	97	98	**98**

**Table 6:** Results of the KNN on multi-classification.

Attacks	Precision %	Recall %	F1 Score %
Analysis	100	100	100
Backdoor	0.00	0.00	0.00
DoS	100	100	100
Exploits	100	100	100
Fuzzers	46	51	48
Generic	99	99	99
Normal	100	100	100
Reconnaissance	58	54	56
Worms	33	0.03	0.05
**Accuracy**	**97.41%**		
**Weighted Average**	**97**	**97**	**97**

**Table 7:** Results of RF on multi-classification.

Attacks	Precision %	Recall %	F1 Score %
Analysis	100	100	100
Backdoor	0.00	0.00	0.00
DoS	100	100	100
Exploits	100	100	100
Fuzzers	49	48	**49**
Generic	99	99	**99**
Normal	100	100	**100**
Reconnaissance	60	59	**59**
Worms	31	11	**16**
**Accuracy**	**97.50%**		
**Weighted Average**	**97**	**98**	**97**

**Table 8:** Results of deep learning using binary classification.

Algorithm	Loss	Accuracy (%)	Precision (%)	Recall (%)	F1 Score (%)
CNN	0.055	98.15	98.98	93.20	96
LSTM	0.0495	98.27	98.28	94.35	96.28

**Table 9:** Results of deep learning using multi-classification.

Algorithm	Loss	Accuracy (%)	Precision (%)	Recall (%)	F1 Score (%)
CNN	0.558	84.46	77	84	80
LSTM	0.30	90.35	88	90	88

**Table 10:** Prediction results of machine learning and deep learning models using a binary dataset.

Model	MAE	MSE	RMSE	R^2^ (%)
SVM	0.0222	0.0222	0.14908	88.11
KNN	0.0189	0.0189	0.1377	89.66
RF	0.01465	0.01465	0.121	92.02
CNN	0.032	0.0147	0.012	91.89
CNN-LSTM	0.0172	0.0172	0.1312	90.50

**Table 11:** Prediction results of machine learning and deep learning model using a multi-classification dataset.

Model	MAE	MSE	RMSE	R^2^ (%)
SVM	0.057	0.157	0.3965	89.39
KNN	0.085	0.1941	0.440	88.92
RF	0.0576	0.156	0.3801	89.35
CNN	0.255	0.533	0.730	65.71
LSTM	0.230	0.717	0.846	90.34

**Table 12:** Results of the proposed system against existing security systems using the same datasets.

Reference	Year	Dataset	Classification Type	Model	Feature Selection	Accuracy (%)
Ref. [75]	2019	UNSW-NB	Multi-classification	SVM	x	75.77%
Ref. [76]	2019	UNSW-NB	Multi-classification	XGBoost	x	86%
Ref. [77]	2019	UNSW-NB	Multi-classification	LSTM	x	83%
Ref. [78]	2019	UNSW-NB	Multi-classification	Dynamic classifier	K-best	61%
Ref. [79]	2021		Binary classification	RF, CNN-LSTM	Information gain Wrapper method application	92.76% 91.91%
Proposed model	2022	UNSW-NB	Multi-classification	SVM, RF	Without using CF	86.23% 89.84%
Proposed model	2022	UNSW-NB	Multi-classification	SVM, RF	Correlation with threshold value of 50%	97.54% 97.50%

## Data Availability

The data presented in this study are available online: https://research.unsw.edu.au/projects/unsw-nb15-dataset (accessed on 15 March 2022).
